# High SARS-CoV-2 infection rates and viral loads in community-dwelling individuals from rural indigenous and mestizo communities from the Andes during the first wave of the COVID-19 pandemic in Ecuador

**DOI:** 10.3389/fmed.2023.1001679

**Published:** 2023-02-09

**Authors:** Diana Morales-Jadán, Alexander Paolo Vallejo-Janeta, Vanessa Bastidas, Maria Belen Paredes-Espinosa, Byron Freire-Paspuel, Ismar Rivera-Olivero, Esteban Ortiz-Prado, Aquiles Rodrigo Henriquez-Trujillo, Tannya Lozada, The UDLA COVID-19 Team, Tatiana Jaramillo, Miguel Angel Garcia-Bereguiain

**Affiliations:** ^1^One Health Research Group, Universidad de las Américas, Quito, Ecuador; ^2^“UDLA COVID-19 Team, ” Universidad de Las Américas, Quito, Ecuador; ^3^Universidad Latina de Costa Rica, San José, Costa Rica

**Keywords:** SARS-CoV-2, indigenous people, COVID-19, Ecuador, Andean region

## Abstract

**Background:**

Neglected indigenous groups and underserved rural populations in Latin America are highly vulnerable to COVID-19 due to poor health infrastructure and limited access to SARS-CoV-2 diagnosis. The Andean region in Ecuador includes a large number of isolated rural mestizo and indigenous communities living under poverty conditions.

**Objective:**

We herein present a retrospective analysis of the surveillance SARS-CoV-2 testing in community-dwelling populations from four provinces in the Ecuadorian Andes, carried out during the first weeks after the national lockdown was lifted in June 2020.

**Results:**

A total number of 1,021 people were tested for SARS-CoV-2 by RT-qPCR, resulting in an overall high infection rate of 26.2% (268/1,021, 95% CI: 23.6–29%), which was over 50% in several communities. Interestingly, community-dwelling super spreaders with viral loads over 10^8^ copies/mL represented 7.46% (20/268, 95% CI: 4.8–11.1%) of the SARS-CoV-2 infected population.

**Conclusion:**

These results support that COVID-19 community transmission in rural communities from the Andean region was happening at the early stages of the COVID-19 pandemic in Ecuador and point out the weakness of the COVID-19 control program. Community-dwelling individuals in neglected rural and indigenous communities should be considered for a successful control and surveillance program in future pandemics in low- and middle-income countries.

## Introduction

Coronavirus disease 2019 (COVID-19), caused by severe acute respiratory syndrome coronavirus 2 (SARS-CoV-2), was first reported in China in December 2019 and spread worldwide, causing the COVID-19 pandemic (1). A few weeks after the initial outbreaks, the first COVID-19 cases were reported in Latin America that has since then been deeply affected. For instance, the first case of COVID-19 was confirmed on 29 February 2020 in Ecuador (2), and during the first year of the COVID-19 pandemic, more than 400,000 COVID-19 cases and 20,000 COVID-19-related deaths have been reported by Ecuadorian public health authorities (3).

Vulnerable groups infected with COVID-19 include not only the elderly and individuals with comorbidities but also historically neglected indigenous populations (4–9). There are more than 476 million indigenous people in the world, highly represented and traditionally neglected in Latin America (10, 11). In Ecuador, indigenous people represent more than 7% of the total population and are mainly associated with underserved rural communities (10–13). Those communities are usually isolated or poorly communicated and have poor access to health services. In many cases, such health services have little capacity and limited coverage, which may delay seeking medical attention, complicating early management, and therefore leading to greater risks of complications and mortality under a scenario such as the COVID-19 pandemic (7–9, 13).

From the early stages of the COVID-19 pandemic, there was a call for action to protect indigenous people from the Americas (7–9). In Ecuador, The National Council for the Equality of Peoples and Nationalities has demanded the protection of indigenous people, reporting COVID-19 outbreaks among their communities and claiming support from public health authorities to contain the pandemic in their communities (8, 11). Moreover, several reports have already shown dramatic SARS-CoV-2 outbreaks leading to community transmission in rural and indigenous populations from the Amazonian and Coastal regions of Ecuador (7, 8, 14–19). Under this scenario, following the request from community leaders, we carried out a SARS-CoV-2 surveillance testing among community-dwelling indigenous and mestizo people in the Ecuadorian Andes few weeks after the population lockdown was lifted in June 2020.

This study aimed to carry out a retrospective analysis of the results of our SARS-CoV-2 testing surveillance in mestizo and indigenous communities from the Ecuadorian Andes to show that COVID-19 community transmission had been happening since the early stages of the pandemic.

## Materials and methods

### Study design and setting

We carried out a retrospective analysis of the data collected from this cross-sectional surveillance to describe the attack rates of SARS-CoV-2 infection among rural indigenous and mestizo communities from the Andean region of Ecuador from June to August 2020. The communities were selected by local public health authorities and community leaders at convenience, using the inclusion criteria of an individual for each household. No random selection of individuals was carried out, so potential bias associated with the sampling cannot be ruled out.

A total of 1,021 community-dwelling individuals were recruited. The communities included in this study belong to the provinces of Chimborazo (communities Lizarzaburu, San Juan, and San Luis at canton Riobamba; community Columbe at canton Colta; and community Penipe at canton Penipe), Tungurahua (communities Benitez, Huambaló, Pelileo, and Salasaca at canton Pelileo), Bolivar (communities Facundo Vela, San Luis, Simiatug, Guaranda, and Veintimilla at canton Guaranda), and Napo (community Oyacachi at canton El Chaco); although Napo is included in the Amazonian provinces of Ecuador, the communities included in this study belong to the highlands area of this province.

In addition, the sociodemographic information was obtained from the official epidemiological record that is mandatory to submit to the local health authority and the Minister of Public Health (MoH) for each sample collected.

### Sample collection, RNA extraction, and RT-qPCR for SARS-CoV-2 diagnosis using the CDC protocol

The samples were processed in the BSL2-certified molecular biology laboratory at Universidad de Las Americas. Nasopharyngeal swabs were collected on a 0.5-mL TE pH 8 buffer for SARS-CoV-2 diagnosis by RT-qPCR, following an adapted version of the CDC protocol as it has been previously described by our laboratory. In brief, the CDC RT-qPCR protocol is based on N1 and N2 probes to detect SARS-CoV-2 and RNase P as an RNA extraction quality control (20–28). In addition, negative controls (TE pH 8 buffer) were included as a control for carryover contamination, one for each set of RNA extractions, to guarantee that only true positives were reported. For viral loads calculation, the 2019-nCoV N positive control (IDT, USA) was used, provided at 200.000 genome equivalents/μL, and a factor of 200 was applied to convert the viral loads to genome equivalents/mL and then converted to a logarithmic scale.

### Statistical analysis

For the statistical analysis of data, infection rates were calculated for each community and province, and also for sex and age group. To assess differences in the infection rates among communities, provinces, sex, or age group, a chi-square test for comparison of proportions was applied. All statistical analyses were carried out using SPSS Statistics 28 software.

## Results

### SARS-CoV-2 infection rates

**Figure 1:**
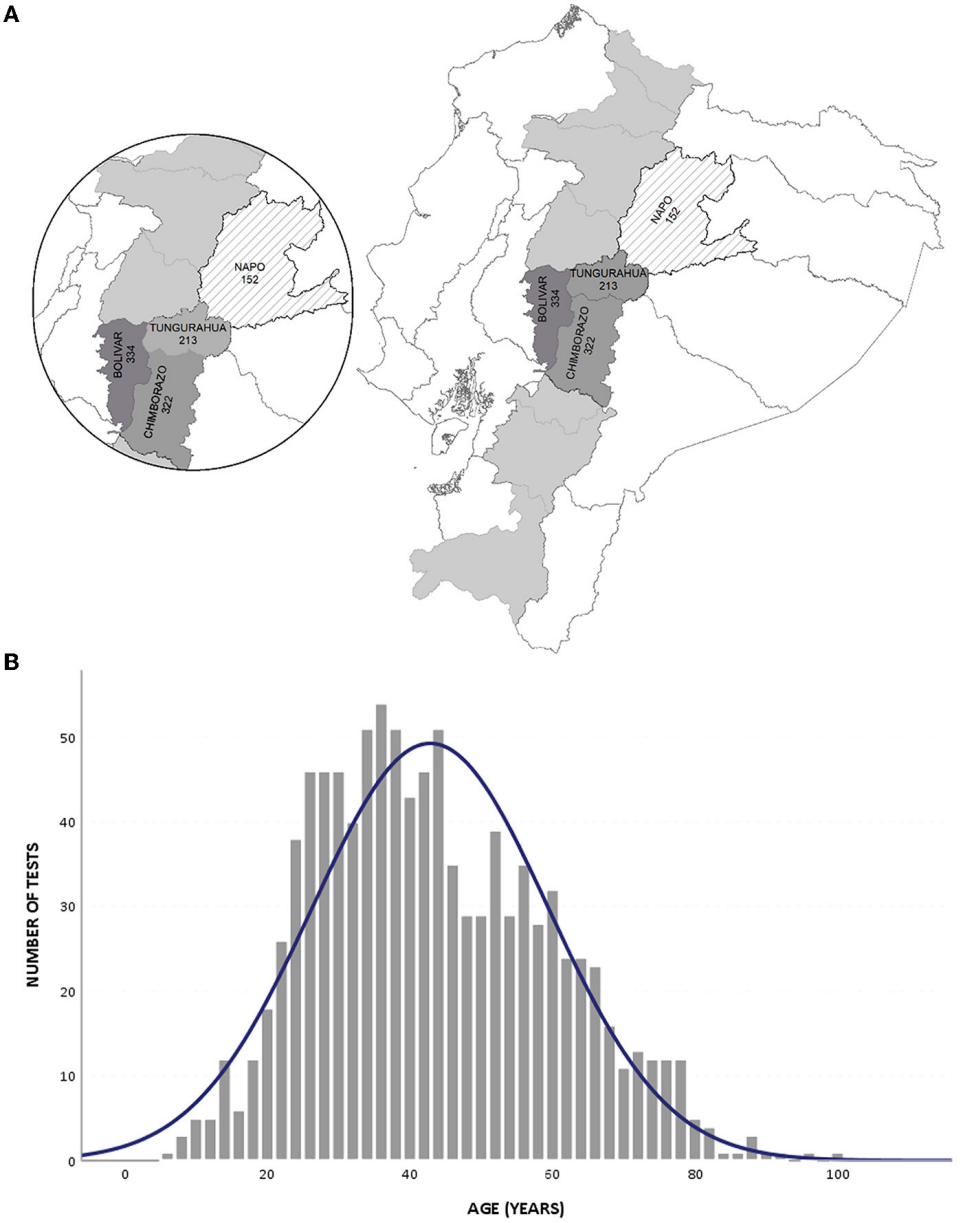
Study population. **(A)** Location of the provinces; the gray area indicates the Ecuadorian Andean region. **(B)** Distribution of tests according to the age of participants.

A total of 1,021 indigenous and mestizo individuals from 15 rural communities distributed along four different provinces of the Ecuadorian Andes were tested for SARS-CoV-2 infection (Figure 1A). For Bolivar province, 334 individuals were recruited, distributed in five locations: Facundo Vela, San Luis, Simiatug, Guaranda, and Veintimilla. For Chimborazo province, 322 individuals were recruited, distributed in five locations: Lizarzaburu, San Juan, San Luis, Columbe, and Penipe. For Tungurahua province, 213 individuals were recruited, distributed in four locations: Benitez, Huambaló, Pelileo, and Salasaca. For Napo province, 152 individuals were recruited from the Oyacachi community. The distribution according to sex was 52.1% (532/1,021) male and 47.9% (489/1,021) female participants. The age distribution for the study population is presented in Figure 1B.

The overall SARS-CoV-2 infection rate was 26.2% (268/1,021, 95% CI: 23.6–29%), with 268 out of 1,021 participants testing positive. The distribution according to sex and age for the individuals infected with SARS-CoV-2 is presented in Table 1. There are no significant differences in the average SARS-CoV-2 infection rate between male and female participants (*p* > 0.05). However, there are significant differences between age groups (*p* < 0.05).

**Table 1:** SARS-CoV-2 infection rates (%) distribution according to sex and age.

	**Sex**		
**Age category years**	**Male**	**Female**	**Total**
Infancy: 0–11	0	1 (0.7%)	1 (0.4%)
Adolescence: 10–18	2 (1.6%)	8 (5.7%)	10 (3.7%)
Youth: 19–26	19 (14.8%)	20 (14.3%)	39 (14.6%)
Adulthood: 27–59	86 (67.2%)	92 (65.7%)	178 (66.4%)
Elderly: more 60	21 (16.4%)	19 (13.6%)	40 (14.9%)
Total	128 (47.8%)	140 (52.2%)	268 (26.2%)

The SARS-CoV-2 infection rates for each province, canton, and community are presented in Table 2. Tungurahua had the highest infection rate value of 139/213, 65.3% (95% IC 58.7–71.4%); followed by Napo with 58/152, 38.2% (95% IC: 30.7–46%); Chimborazo with 54/322, 16.77% (95% IC: 12–21.6%); and Bolivar with 17/334, 5.1% (95% IC: 3.1–7.8%). The SARS-CoV-2 infection rates for cantons comprised Guaranda 5.09% (17/334), Colta 12% (4/33), Penipe 20.6% (13/63), Riobamba 16.4% (37/226), El Chaco 38.2% (58/152), and Pelileo 65.3% (139/213). The SARS-CoV-2 infection rates for communities comprised Simiatug 7.9% (3/38), Veintimilla 7.4% (5/68), Guaranda 4.6% (8/175), San Luis 3.7% (1/27), Facundo 0% (0/26), Columbe 12% (4/33), Penipe 21% (13/63), San Juan 19 % (26/136), San Luis 13% (9/68), Lizarzaburu 9% (2/22), Oyacachi 38% (58/152), Huambaló 74% (58/78), Salasaca 68% (46/68), Benitez 64% (18/28), and Pelileo 44% (17/39). Significant differences were found between those values (*p* < 0.01).

**Table 2:** SARS-CoV-2 infection rates for each province, canton, and community included in this study.

**Province**	**Canton**	**Community**	**Positive/** **total;** **infection** **rate (%)**	**Overall** **infection** **rate (%)**
Bolívar	Guaranda	Facundo	0/26; 0%	17/334; 5.1% (95% IC: 3.1–7.8%)
		Guaranda	8/175; 4.6%	
		San Luis	1/27; 3.7%	
		Simiatug	3/38; 7.9%	
		Veintimilla	5/68; 7.4%	
Chimborazo	Colta	Columbe	4/33; 12.1%	54/322; 16.77% (95% IC: 12–21.6%)
	Penipe	Penipe	13/63; 21%	
	Riobamba	Lizarzaburu	2/22; 9%	
		San Juan	26/136; 19%	
		San Luis R	9/68; 13%	
Napo	El Chaco	Oyacachi	58/152	58/152; 38.2% (95% IC: 30.7–46%)
Tungurahua	Pelileo	Benítez	18/28; 64.3%	139/213; 65.3% (95% IC 58.7–71.4%)
		Huambaló	58/78; 74%	
		Pelileo	17/39, 44%	
		Salasaca	46/68; 68%	
		Overall	268/1021; 26.2%	

### SARS-CoV-2 viral loads

**Figure 2:**
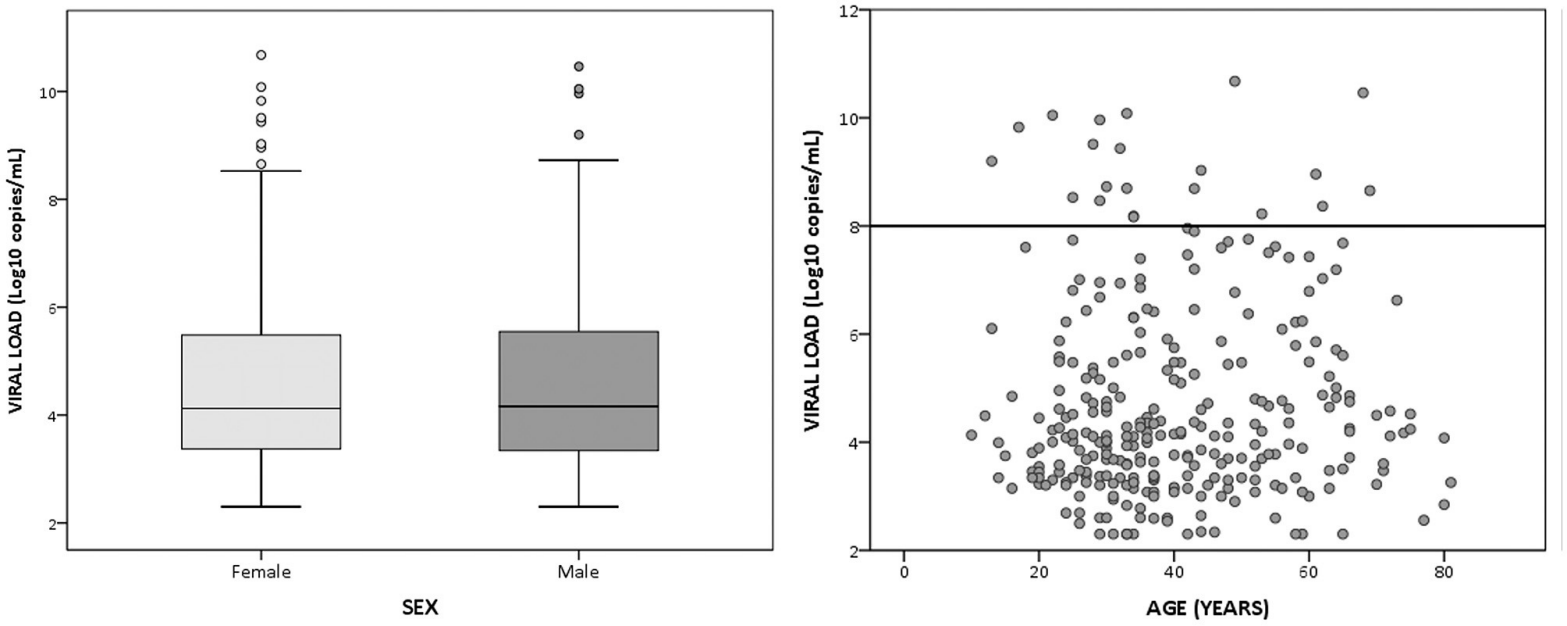
Distribution of SARS-CoV-2 viral loads according to sex and age in the study population. Viral load is represented in a log scale.

The distribution of SARS-CoV-2 viral loads according to sex and age is presented in Figure 2. No significant differences were found (*p* > 0.05). In addition, 20 individuals had viral SARS-CoV-2 load values of above 10^8^ copies/mL belonging to the cantons of Penipe (3), Riobamba (5), El Chaco (7), and Pelileo (5). Those individuals represented 7.46% (20/268, 95% CI: 4.8–11.1%) of the individuals infected with SARS-CoV-2.

## Discussion

Due to the retrospective nature of this study, there was not a randomized sample collection to include a statistically representative population sampling for these provinces in the Andean region. This is a strong limitation in our study, as the bias on sample collection could mean that the results obtained were not truly representative of the COVID-19 epidemiological context in this region but were limited to the communities selected. However, as the average SARS-CoV-2 infection rate was over 26% (peaking over 50% in several communities) and outbreaks were found at 14 out of 15 communities visited, our results would suggest that non-control COVID-19 community transmission had been happening among rural indigenous communities in the Andes just a few weeks since the national lockdown was lifted. It has been reported that the current health crisis caused by COVID-19 has further aggravated the conditions of vulnerability and social exclusion of indigenous populations in Latin America, and the Andean region would not be an exception (29–33). Similarly, severe COVID-19 outbreaks have been described for Amazonian indigenous people in Brazil and Ecuador (7–9) despite the supposed isolation of those ethnic groups, pointing out the high vulnerability to COVID-19 of those traditionally neglected communities (29–36). In addition, rural communities from the Coastal Region of Ecuador in the provinces of Esmeraldas, Manabí, and Santa Elena were deeply affected by COVID-19 outbreaks during the first wave of the pandemic (14–16, 19). Although widespread, the COVID-19 pandemic has burdened neglected rural and indigenous populations more than others due to limited access to water, poor sanitation of households, lack of information in indigenous languages, and limited access to the healthcare system (32–36).

Interestingly, this study included only community-dwelling non-hospitalized individuals, so either no symptoms or mild symptoms were reported among the individuals infected with SARS-CoV-2. Moreover, 20 individuals from four different cantons had viral loads over 10^8^ viral copies/mL and could be considered SARS-CoV-2 super spreaders, representing a 7.46% of the infected population (37). Although there are limitations associated with the calculated viral load based on Ct values representing all the viral genomic material on the sample, and infection of cell cultures is used for sample infectivity confirmation, it is a clear association between low Ct values (that indicates high viral loads based on genomic material quantification) and infectivity (37). As the COVID-19 control and surveillance program in Ecuador was mainly limited to hospitalized individuals, our results clearly endorsed that the strategy was not sufficient to control COVID-19 outbreaks (14–19). Nevertheless, the SARS-CoV-2 testing capacity for the public health system in Ecuador was very limited for a 17-million population (38–40). In addition, no resources were allocated to most of the rural provinces of the country, and the SARS-CoV-2 diagnosis was centralized in the three laboratories from the “Instituto Nacional de Salud Publica e Investigación” located in the three main cities of Ecuador (18). Together with studies carried out in Afro-Ecuadorian communities (19), rural villages from the Manabi province (14, 15), Amazonian indigenous communities (7, 8), women victims of gender-based violence (41), food riders, or funeral home workers (42, 43) from Ecuador, those results highlight the need for active COVID-19 monitoring in community-dwelling individuals from vulnerable groups and neglected communities.

In conclusion, our findings support that COVID-19 community transmission and super-spreading events were happening among rural mestizo and indigenous communities from the Andean region in Ecuador during the first wave of the COVID-19 pandemic. For the still ongoing COVID-19 pandemic and future ones, our results endorse that control and prevention strategies have to focus not only on hospitalized and symptomatic individuals but also on community-dwelling individuals at locations where outbreaks are suspected.

## Author's note

In this study, we described COVID-19 outbreaks in rural indigenous population from the Andean Region of Ecuador. Although several studies regarding COVID-19 and indigenous people have been published from Latin America, this is the first one addressing the situation in the Andean region during the early stages of the pandemics. With a sample over 1,000 community dwelling individuals, high infection rates were found endorsing community transmission during the first wave of COVID-19 pandemic in these neglected population in Ecuador.

## Data availability statement

The original contributions presented in the study are included in the article/supplementary material, further inquiries can be directed to the corresponding author.

## Ethics statement

The paired samples used for the homogenization protocol were the leftover of the samples collected for routine SARS-CoV-2 diagnosis. Nevertheless, this work is included in a study that was approved by the IRB from the Dirección Nacional de Inteligencia de la Salud (Ministerio de Salud Publica, Ecuador) under the code 008-2020.

## UDLA COVID-19 team

Tatiana Jaramillo, Daniela Santander Gordon, Gabriel Alfredo Iturralde, Julio Alejandro Teran, Karen Marcela Vasquez, Jonathan Dario Rondal, Genoveva Granda, Ana Cecilia Santamaria, Cynthia Lorena Pino, Oscar Lenin Espinosa, Angie Buitron, David Sanchez Grisales, Karina Beatriz Jimenez, Heberson Galvis, Barbara Coronel, Dayana Marcela Aguilar, Ines Maria Paredes, Christian David Bilvao, Sebastian Rodriguez Pazmiño, Juan Carlos Laglaguano, Henry Herrera, Pablo Marcelo Espinosa, Edison Andrés Galarraga, Marlon Steven Zambrano-Mila, Ana María Tito, and Nelson David Zapata.

## Author contributions

DM-J and MG-B wrote the manuscript. All authors listed have made a substantial, direct, and intellectual contribution to the work and approved it for publication.
